# Hippocampal structural alterations in early-stage psychosis: Specificity and relationship to clinical outcomes

**DOI:** 10.1016/j.nicl.2022.103087

**Published:** 2022-06-16

**Authors:** Gina Brunner, Ruchika Gajwani, Joachim Gross, Andrew I. Gumley, Rajeev Krishnadas, Stephen M. Lawrie, Matthias Schwannauer, Frauke Schultze-Lutter, Alessio Fracasso, Peter J. Uhlhaas

**Affiliations:** a Institute for Neuroscience and Psychology, Univ. of Glasgow, UK; b Institute of Health and Wellbeing, Univ. of Glasgow, UK; c Institute of Biomagnetism and Biosignalanalysis, University of Muenster, Muenster, Germany; d Department of Psychiatry, Univ. of Edinburgh, UK; e Department of Clinical Psychology, Univ. Edinburgh, UK; f Department of Psychiatry and Psychotherapy, Medical Faculty, Heinrich Heine University, Düsseldorf, Germany; g Department of Psychology, Faculty of Psychology, Airlangga University, Airlangga, Indonesia; h University Hospital of Child and Adolescent Psychiatry and Psychotherapy, University of Bern, Switzerland; i Department of Child and Adolescent Psychiatry, Charité Universitätsmedizin, Berlin, Germany

**Keywords:** Hippocampus, Psychosis, Clinical high risk, Subcortex, Morphometry

## Abstract

Hippocampal dysfunctions are a core feature of schizophrenia, but conflicting evidence exists whether volumetric and morphological changes are present in early-stage psychosis and to what extent these deficits are related to clinical trajectories. In this study, we recruited individuals at clinical high risk for psychosis (CHR-P) (n = 108), patients with a first episode of psychosis (FEP) (n = 37), healthy controls (HC) (n = 70) as well as a psychiatric control group with substance abuse and affective disorders (CHR-N: n = 38). MRI-data at baseline were obtained and volumetric as well as vertex analyses of the hippocampus were carried out. Moreover, volumetric changes were examined in the amygdala, caudate, nucleus accumbens, pallidum, putamen and thalamus. In addition, we obtained follow-up functional and symptomatic assessments in CHR-P individuals to examine the question whether anatomical deficits at baseline predicted clinical trajectories. Our results show that the hippocampus is the only structure showing significant volumetric decrease in early-stage psychosis, with FEPs showing significantly smaller hippocampal volumes bilaterally alongside widespread shape changes in the vertex analysis. For the CHR-P group, volumetric decreases were confined to the left hippocampus. However, hippocampal alterations in the CHR-P group were not robustly associated with clinical outcomes, including the persistence of attenuated psychotic symptoms and functional trajectories. Accordingly, our findings highlight that dysfunctions in hippocampal anatomy are an important feature of early-stage psychosis which may, however, not be related to clinical outcomes in CHR-P participants.

## Highlights

• Individuals with early-stage psychosis show reduced hippocampal volumes.
• FEP show bilateral and widespread changes, while left hemisphere is affected in CHR-P.
• However, hippocampal changes do not show a relationship with clinical outcomes.

## 1 Introduction

Psychotic disorders, such as schizophrenia (ScZ), have been associated with neuroanatomical changes, including grey matter (GM) alterations in cortical (Glahn et al., 2008) and subcortical regions (van Erp et al., 2016, Gutman et al., 2022), that have been related to negative symptoms (Walton et al., 2018) as well as to cognitive deficits (Pantelis and Nelson, 2019). In addition to cortical changes, reductions in the hippocampus, amygdala, thalamus and nucleus accumbens have been observed, while pallidum volume is increased in ScZ (van Erp et al., 2016). Volumetric changes in subcortical areas correlate with surface alterations that include both surface contractions and increases (Gutman et al., 2022).

Recent work has investigated the role of GM changes in participants at clinical high risk for psychosis (CHR-P) to identify biomarkers for early detection and prognosis (Jalbrzikowski et al., 2021). There is extensive evidence that ScZ is preceded by a prodromal phase of up to 5 years (Fusar-Poli et al., 2020a, Fusar-Poli et al., 2020b, Klosterkötter et al., 2001) that involves subtle alterations in cognition and functioning that could be mediated by changes in GM (Koutsouleris et al., 2010). Reductions in cortical GM have been identified in CHR-P individuals (Group, Jalbrzikowski et al., 2021, Zikidi et al., 2020), which may be related to transition to psychosis (Koutsouleris et al., 2009) as well as functional outcomes (Koutsouleris et al., 2018). Relationships with clinical outcomes are a particularly important issue as only a minority of CHR-P participants, approximately 25% over a three-year period (Pablo et al., 2021), will eventually transition to a first-episode of psychosis (FEP). While there is consistent evidence for cortical GM changes in CHR-P participants, evidence for a possible contribution of subcortical regions in emerging psychosis is less clear.

A region that has received particular attention during early-stage psychosis is the hippocampus (Provenzano et al., 2020). Previous studies suggested that abnormal functioning and anatomy of the hippocampus may constitute one of the earliest signs of psychosis (Lieberman et al., 2018). Specifically, it has been proposed that dysregulated neurotransmission of glutamatergic circuitry may lead to excitotoxic effects (Lisman et al., 2008) and abnormal hippocampal activation (Allen et al., 2015, Allen et al., 2021, Modinos et al., 2020a, Modinos et al., 2020b), resulting in volumetric reductions (Provenzano et al., 2020). Moreover, these changes may in turn drive functional and structural abnormalities in dopaminergic neurotransmission (Modinos et al., 2021, Stone et al., 2010), indicating that the hippocampus could play a key role in the pathophysiology of ScZ by triggering a cascade of events leading to widespread cortical and subcortical circuit changes. The subregion CA1 has received particular attention (Schobel et al., 2013), but there is also evidence for abnormalities in CA2 and CA3 (Baglivo et al., 2018).

Despite the prominent role of the hippocampus in ScZ, there is currently conflicting evidence whether hippocampal alterations are present in early-stage psychosis. In CHR-P participants, some studies have reported intact hippocampus volumes (Walter et al. 2016, 2020), while others have reported overall volumetric reductions (Ganzola et al., 2014, Harrisberger et al., 2016a, Harrisberger et al., 2016b, Harrisberger et al., 2016c, Wood et al., 2010), in particular in CA1 (Lieberman et al., 2018). Similarly, hippocampal hyperactivity, as reflected by elevated blood flow and glutamate levels, predicted transition to psychosis in CHR-P participants (Bossong et al., 2019, Provenzano et al., 2020). In contrast, the majority of studies in FEP-patients have reported hippocampal reductions (Adriano et al., 2012, Borgwardt et al., 2007, Buehlmann et al., 2010, Lieberman et al., 2018, Phillips et al., 2002, Velakoulis et al., 2006), suggesting the possibility of progressive dysfunctions with illness stages.

To clarify the role of the hippocampus in early-stage psychosis, we performed volumetric and morphological analyses of the hippocampus and other subcortical structures (amygdala, caudate, nucleus accumbens, palladium, putamen, thalamus) in CHR-Ps and FEP-patients. This is because it is currently unclear whether anatomical alterations are specific to the hippocampus or whether subcortical regions, such as the nucleus accumbens, caudate (Sasabayashi et al., 2020), and thalamus (Harrisberger et al., 2016a), are also affected. Moreover, antipsychotic and antidepressant medication (APM/ADM) have previously been shown to affect subcortical volumes (Hashimoto et al., 2018). Accordingly, we also tested the effects of APM/ADM on anatomical variables in CHR-P and FEP-groups.

We also included a group of participants with affective and substance use disorders who did not meet CHR-P criteria (CHR-N) in addition to non-clinical control participants (HC). There is evidence that hippocampal changes also occur in several other psychiatric syndromes, including major depressive disorder (Arnone et al., 2012) as well as substance abuse (Wilson et al., 2017, Wang et al., 2021) and there is substantial comorbidity between affective disorders, substance abuse and early-stage psychosis (Li et al., 2020, Wilson et al., 2017, Herniman et al., 2021). Finally, we investigated the relationship between hippocampal volumes and clinical features, including global functioning and cognition, and the persistence of attenuated psychotic symptoms (APS) to determine whether hippocampal changes correlate with clinical and functional outcomes in CHR-P participants.

## 2 Materials and methods

### 2.1 Participants

A total of 253 participants were recruited from the Youth Mental Health Risk and Resilience (YouR) Study (Uhlhaas et al., 2017) and divided into four groups: 1) 108 participants meeting CHR-P criteria, (2) 38 participants characterized by non-psychotic disorders, such as affective disorders (n = 11), anxiety disorders (n = 16), eating disorders (n = 1), and/or substance abuse (n = 10) (CHR-N), 3) 37 patients with FEP (15 antipsychotic-naïve) and, 4) 70 healthy control participants (HC) without an axis I diagnosis or family history of psychosis. Ages across groups ranged from 16 to 34 years.

CHR-P status at baseline was established by ultra-high risk criteria according to the Comprehensive Assessment of At Risk Mental States (CAARMS) Interview (Yung et al., 2005) and the Cognitive Disturbances (COGDIS) and Cognitive-Perceptive (COPER) basic symptoms criteria according to the Schizophrenia Proneness Instrument, Adult version (SPI-A (Schultze-Lutter et al., 2012). FEP patients were assessed with the Structured Clinical Interview for DSM-5 (SCID, First, 2014) and with the Positive and Negative Symptom Scale (PANSS, Kay et al., 1987). For all groups except FEP-patients, cognition was assessed with the Brief Assessment of Cognition in Schizophrenia (BACS) (Keefe et al., 2004).

The study was approved by the ethical committees of University of Glasgow and the NHS Research Ethical Committee Glasgow & Greater Clyde. All participants provided written informed consent.

### 2.2 MRI acquisition

We acquired T1-weighted MR images on a 3 T Siemens scanner using a 3D MPRAGE sequence with the following parameters: FoV: 256 × 256 × 176 mm3, voxel size: 1 × 1 × 1 mm3, TR: 2250 ms, TE: 2.6 ms, TI: 900 ms, FA: 9°.

#### 2.2.1 Preprocessing

Pre-processing was performed using ANTs (http://stnava.github.io/ANTs/), AFNI (https://afni.nimh.nih.gov/), FSL (https://fsl.fmrib.ox.ac.uk/fsl/fslwiki) and custom functions in R (https://www.r-project.org/). DICOM images were converted to nifti (.nii) files using the function dcm2niix_afni. T1-w volumes were up-sampled to 0.8 mm isotropic. Up-sampling was performed using the AFNI function 3dresample, using linear interpolation. Single-participant volumes were skull-stripped using the afni function 3dSkullStrip. The intensity of T1-w volumes was normalized to remove global inhomogeneities using the ANTs function N4BiasFieldCorrection. Volumes were normalized to Talairach space using the function @auto_tlrc and the corresponding affine transformations were stored for subsequent use. FSL FIRST was used to extract subcortical segmentations for the following structures: thalamus, putamen, pallidum, caudate, amygdala, hippocampus and nucleus accumbens. Lastly, we transformed the result of FSL segmentation back into the original single-participant space by inverting the affine transformation. The computation of subcortical volume was performed in the original single-participant space. We also obtained an estimate of total brain volume (TBV) from the brain mask obtained from 3dSkullStrip.

**Fig. 1:**
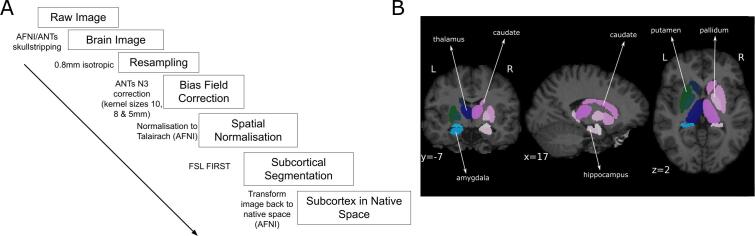
Analysis pipeline and example segmentation outcome from FSL FIRST. Panel A: flowchart reporting the preprocessing steps using AFNI and ANTs functions. B: subcortical segmentation example obtained from a single participant. The quality of all segmented images was determined by visual inspection.

It is important to note that although Freesurfer has shown greater consistency with manual segmentations in pediatric and longitudinal data (Cover et al., 2016, Schoemaker et al., 2016), FSL-FIRST has consistently outperformed the non-longitudinal Freesurfer pipeline on hippocampal (Cover et al., 2016, Næss-Schmidt et al., 2016, Velasco-Annis et al., 2018) and other subcortical structures (Perlaki et al., 2017). We therefore chose FSL-FIRST to perform subcortical segmentations (see in Fig. 1). All images were visually inspected, whereby images with visible artifacts and poor quality segmentations (i.e. visibly inconsistent with how the image would have been segmented manually) were excluded. Data from one CHR-P individual was excluded from all analyses due to motion artifacts.

### 2.3 Volumetric analysis

Volumetric measurements were extracted from the FSL segmentations and averaged across the two hemispheres for the following structures: thalamus, putamen, pallidum, caudate, amygdala, hippocampus, nucleus accumbens. We conducted a GLM analysis which tested for differences in volumes between HC and each clinical group with age and TBV being used as covariates. The following equation was used in R:

Subcortical volume∼Group+TBV+age

The R function aov was additionally used to identify main effects of group. FDR correction was applied to correct for multiple comparisons; all analyses were conducted using the software R and the lme4 package. Analyses were also conducted for hemispheres and structures separately while controlling for years of education and handedness (Supplementary Table 1). In addition, we tested for the effects of antidepressant medication in the CHR-Ps and antipsychotic medication in the FEP groups on subcortical volumes (Supplementary Tables 4 and 5).

### 2.4 Vertex analysis

Following our volumetric analysis, we used FSL to examine regional shape differences. While FSL does not directly extract subfield volumes, subfield-specific changes can be inferred from the location of shape deformations on the surface of each structure. Vertex analysis therefore allows us to determine where potential volumetric changes from the prior volumetric analysis likely originated from – i.e. whether they are regionally specific or widespread across a given subcortical structure. Surface meshes were extracted for each participant from the subcortical segmentations generated using FSL FIRST. Design matrices were generated for each pairwise group comparison and hemisphere separately.

We used the same covariates as for the linear models (i.e. total brain volume, age); covariate scores were mean-centered for the vertex analysis. FSL randomize was then used to generate 10,000 permutations (per comparison), and to compute the F-statistic and significance (FWE-corrected at the cluster level at a minimum cluster size of 3 voxels). Bonferroni-correction was additionally applied to correct for the multiple comparisons between groups conducted.

For visualization purposes, the generated masks showing F-values were plotted in MNI space using the Python package nilearn. The masks were thresholded to only show F-values where the cluster p-value ≤ 0.05; analyses which returned no significant clusters were omitted from plotting.

### 2.5 Clinical Follow-Up

Participants meeting CHR-P criteria were reassessed at 3-, 6-, 9-, 12-, 18-, 24-, 30-, and 36-month intervals to examine the persistence of APS and functional outcomes, using the CAARMS interview. Based on past research (Allen et al., 2015; Modinos et al., 2019), GAF outcome categories were split into good (GFO) and poor functional outcomes (PFO) using a cutoff of GAF ≥ 65. For the follow-up analyses, we used GAF data from 6- and 12-months follow-ups.

Persistence of ultra-high risk criteria was operationalized by the continued presence of APS up to 12 months. In addition, transition to psychosis was assessed. We fitted binomial GLMs for each clinical outcome (i.e. APS persistence, functional outcomes, transition to psychosis) in the CHR-P group to investigate the relationship between hippocampal volumes and outcomes, using the same covariates (TBV, age) as our aforementioned linear models with the R package lme4.

## 3 Results

### 3.1 Demographic and clinical data

In the CHR-P group, n = 30 individuals showed persistent APS and n = 10 transitioned to psychosis (mean follow-up period to transition: 19.2 months). N = 78 CHR-Ps were characterized by GAF scores < 65 at baseline, n = 57 at 6 months follow-up, and n = 40 at 12-months follow-up. The groups showed differences in gender and age distribution, whereby the FEP group was slightly older and included more male participants. CHR-P individuals additionally showed significantly lower GAF, motor speed and total BACS scores than HC individuals (see Table 1).

**Table 1:** Demographical and Clinical Data.

	HC (N = 70)	CHR-N (N = 38)	CHR-P (N = 108)	FEP (N = 37)	Group effect	Post-hoc comparisons
**Age** (M, SD)	23.59 (3.87)	22.95 (4.66)	21.81 (4.46)	24.76 (4.15)	F = 7.06, p <.001	HC < CHR-P FEP > CHR-P
**Gender** (F, %)	39 (55.71)	26 (68.42)	80 (74.07)	15 (40.54)	χ = 17.05, p <.001	
**Education** (years)	16.65 (3.05)	16.46 (3.45)	15.30 (3.21)	16.20 (3.30)	F = 2.58, p =.054	
**Medication** (n, %)				*		
None	70 (1 0 0)	23 (60.52)	50 (46.30)	4 (26.67)	–	–
Antidepressant	–	8 (21.05)	33 (30.56)	3 (20)	–	–
Antipsychotic	–	0 (0)	1 (0.92)	6 (40)	–	–
Other	–	7 (18.42)	30 (27.78)	7 (46.67)	–	–
**CAARMS severity**				*		
Total score (M, SD)	–	6.18 (6.21)	30.29 (4.64)	–	F = 45.33, p <.01	CHR-P > CHR-N CHR-P > HC
UTC	–	0.61 (1.15)	1.84 (1.93)	–	–	–
NBI	–	0.79 (1.04)	2.91 (1.76)	–	–	–
PA	–	0.97 (1.35)	2.87 (1.50)	–	–	–
DS	–	0.52 (0.89)	1.42 (1.38)	–	–	–
**CHR category**						
CAARMS only (APS/GFRD)	–	–	31	–	–	–
SPI-A only (COGDIS/COPER)	–	–	29	–	–	–
CAARMS + SPI-A	–	–	51	–	–	–

	**HC (N = 70)**	**CHR-N (N = 38)**	**CHR-P (N = 108)**	**FEP (N = 37)**	**Group effect**	**Post-hoc comparisons**

**BACS**						

Composite score	0.21 (0.78)	−0.05 (1.59)	−0.64 (1.67)	–	F = 3.59, -p =.03	HC > CHR-P
Verbal memory	0.25 (1.0)	0.20 (1.73)	0.01 (1.27)	–	F = 0.524, p =.59	–
Verbal fluency	0.07 (1.54)	−0.24 (1.01)	−0.03 (1.16)	–	F=,0.58p =.56	–
Working memory (Digit sequencing)	0.19 (1.0)	0.29 (1.26)	0.05 (1.36)	–	F = 0.39, p =.68	–
Motor speed (Token task)	0.0 (0.97)	−0.70 (1.0)	−1.10 (1.41)	–	F = 9.60p. < 0.01	HC > CHR-P
Executive functioning (Tower of London)	0.10 (0.79)	0.24 (1.25)	−0.16 (1.41)	–	F=,1.41p =.32	HC > CHR-P
GAF0 (M, SD)	87.57 (6.49)	70.05 (12.76)	58.33 (13.83)	–	F = 79.82, p <.01	HC > CHR-N HC > CHR-P CHR-N > CHR-P
GAF6 (M, SD)	–	57.73 (20.3)	58.8 (13.71)	–	–	
GAF12 (M, SD)	–	66.59 (20.32)	62.59 (14.52)	–	–	
N at follow-up (6 m, 12 m)	–	15, 20	88, 74	–	–	

**Abbreviations:** APS, attenuated psychotic symptoms; BACS, Brief Assessment of Cognition in Schizophrenia; CAARMS, Comprehensive Assessment of At Risk Mental States; COGDIS, Cognitive Disturbances, COGDIS, Cognitive-Perceptive Basic Symptoms criterion; HC, healthy HCtrols; CHR-N, clinical risk-negative; CHR-P, clinical high-risk positive; FEP, first-episode psychosis; GAF, global assessment of functioning; SPI-A, Schizophrenia Proneness Instrument, Adult version; SD, standard deviation of the mean; AD, antidepressant; AP, antipsychotic.

**Note**: * data only available for 15 participants.

### 3.2 Volumetric analysis

We conducted a general linear model (GLM) analysis for each subcortical structure. A significant effect of TBV was observed, indicating a positive scaling between subcortical structures volume and TBV (t-values ranging between 5.97 for the amygdala to 18.14 for the thalamus, all p > 0.05, Bonferroni corrected). An effect of age was also observed for the thalamus and the hippocampus, indicating a positive scaling between volume and age (t of 3.56 and 2.95, respectively, p <.05, Bonferroni corrected).

**Fig. 2:**
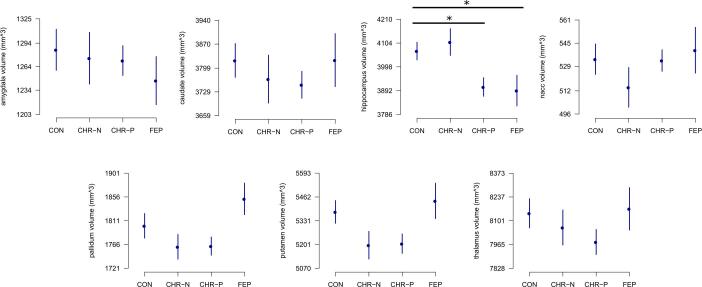
Volumetric analysis, results. Average volumetric results for each subcortical structure across the control group (HC), clinical controls (CHR-N), clinical high-risk (CHR-P) and first-episode psychosis (FEP). Error bars indicate ±1 standard error of the mean (sem). Volumetric results are reported in cubic millimeters. *** indicate a significant difference between the groups, Bonferroni corrected, p < 0.05.

We observed a main effect of group on hippocampal volumes (F = 5.67, *p* <.01). A significant, bilateral reduction in hippocampal volume was found for FEPs vs. HCs (t = −3.75, p <.05, FDR corrected) (Fig. 2, Supplementary Table 1). The difference between CHR-Ps and HCs also was significant (t = −2.38, p =.017) before but not after FDR correction (p =.06). A reduction in volume was observed in the left (t = −2.69, p = 0.008, uncorrected) but not in the right hippocampus (t = −1.58, p = 0.116, uncorrected) in the CHR-P group compared to HCs. The difference between FEP and CHR-P groups was not significant (t = 1.8, p =.08).

No other subcortical structures were characterized by significant differences between clinical groups and HC (Fig. 2). Differences in the FEP group in the thalamus and amygdala as well as in the putamen in the CHR-P group showed p <.1, but were all nonsignificant at p <.05 after FDR correction (see Supplementary Table 1).

### 3.3 Vertex analysis

**Fig. 3:**
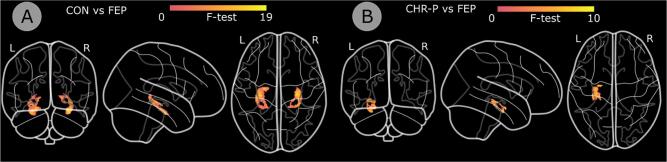
Vertex analysis at the level of the hippocampus. Hippocampal masks output by FSL showing the values of the F-statistic are overlaid onto a 1 mm MNI standard image. Panel A: the comparison between patients with a first-episode of psychosis (FEP) and healthy controls (HC) revealed significant shape differences. Panel B: the comparison between the clinical high-risk (CHR-P) and first episode (FEP) revealed significant shape differences in the anterior to mid-left hippocampus. No differences were observed in the right hemisphere.

Vertex analysis was limited to the hippocampus since this was the only subcortical structure that differed between groups (Fig. 3). The HC and FEP groups showed significant bilateral differences across the hippocampal surface (Peak cluster left: voxel 62, 106, 50 (MNI152 1 mm; p =.001, F = 16.12); peak cluster right: 116, 105, 50 (p =.0004, F = 18.91). In the CHR-P group, clusters with the highest F-values were primarily concentrated around the most anterior and posterior hippocampus in both hemispheres. However, no differences between CHR-P and HC, or CHR-P and CHR-N groups were significant (Supplementary Table 3).

For the FEP and CHR-P groups, there was a significant difference in the anterior to mid-left hippocampus (p =.007, F = 10.17), with a peak cluster around voxel 120, 103, 61 (Fig. 3). No differences were observed in the right hemisphere (see Supplementary Table 3).

### 3.4 Correlations with cognition and clinical measures

Hippocampal volumes did not show any significant correlations with global functioning (GAF), cognition (BACS total score and subscales), and symptom severity (CAARMS total and subscales, SPI-A severity) in the CHR-P group (see Supplementary Table 2). A relationship, however, was observed between left hippocampal volumes and GAF score at 12 months follow-up, but this was not significant after correction for multiple comparisons.

### 3.5 Subcortical volumes and clinical outcomes in CHR-P participants

We compared hippocampal volumetric data for CHR-P participants who continued to meet criteria for persistent APS at 12-month follow-up (APS-P: n = 32; APS-NP: n = 40). There were no significant differences between CHR-P subgroups (p =.14, t = -1.49). Moreover, a binomial GLM did not reveal a significant relationship between hippocampal volume and transition to psychosis (p >.10, see Supplementary Table 6).

In addition, the relationship with good and poor functional outcomes at baseline as well as at 6- and 12-months follow-up using logistic regression were explored. Mean hippocampus volume (averaged across hemispheres) did not show a significant association with GAF category at baseline (*p* =.58, uncorrected) or at 6 months (*p* =.33, uncorrected), but a relationship with GAF category at 12 months was detected (β = 0.0018, *p* =.036, Bonferroni-corrected) (Supplementary Table 6).

### 3.6 Medication effects on subcortical volumes

In the CHR-P group, ADM medication status did not show a significant relationship with any subcortical volumes in either hemisphere (all p > 0.1, uncorrected), including the hippocampus. Similarly, no effect was found in the FEP group for APM-status (Supplementary Table 5).

## 4 Discussion

The current study examined alterations in hippocampal volume and morphology during early-stage psychosis to address the specificity of hippocampal changes, relationship to illness stage as well as the link with clinical outcomes in CHR-P participants. We detected hippocampal volume-reductions in both CHR-P and FEP groups which were not present in psychiatric controls nor was any other subcortical structure characterized by anatomical deficits. Hippocampal volumes did not, however, robustly predict clinical and functional outcomes in CHR-P participants.

There is currently inconsistent evidence for hippocampus alterations in CHR-Ps. Although several studies have observed reduced hippocampus volumes (Borgwardt et al., 2007, Ganzola et al., 2014, Harrisberger et al., 2016a, Harrisberger et al., 2016b, Harrisberger et al., 2016c, Sasabayashi et al., 2021, Wood et al., 2010), recent meta-analyses (Walter et al., 2016, Hinney et al., 2021) observed no robust evidence for volumetric reductions.

Reduced hippocampus volumes have been previously shown to predict transition to psychosis (Buehlmann et al., 2010, Provenzano et al., 2020, but see Hinney et al., 2020) as well as a persistence of APS (Ho et al., 2016, Ho et al., 2017a, Ho et al., 2017b), especially in the hippocampal subregion CA1. In the current study, hippocampal volumes did not differ between CHR-P with persistent vs. non-persistent APS nor were CHR-Ps who transitioned to psychosis characterized by exaggerated GM-reductions. However, there was a nonsignificant association between hippocampal volumes and GAF at 12 months but not at 6 months in the CHR-P group. Given the smaller number of follow-up data for CHR-Ps at 12 months, one possibility is that this effect is driven by attrition of participants.

In FEP patients, more robust deficits have been reported for both hippocampal volume and shape (Adriano et al., 2012, Borgwardt et al., 2007, Buehlmann et al., 2010, Lieberman et al., 2018, Phillips et al., 2002, Velakoulis et al., 2006), particularly in the anterior portion (McHugo et al., 2020; see also Haukvik et al., 2016). Interestingly, the extent of volume loss may be linked with the duration of untreated psychosis (Briend et al., 2020) and reduced hippocampal volume might be prognostic for clinical outcomes (McHugo et al., 2020).

In the current study, volumetric reductions in the FEP-group involved the bilateral hippocampus while in CHR-P participants deficits were confined to the left hemisphere. There is inconsistent evidence for the role of hemispheric differences in the hippocampus in early-stage psychosis. Some have observed changes in the left hemisphere that were associated with transition to psychosis (Buehlmann et al., 2010), FEP status (Baglivo et al., 2018, Velakoulis et al., 2006) and illness chronicity (Sasabayashi et al., 2021), while others did not report hemispheric differences in CHR-Ps (Harrisberger et al., 2016a, Harrisberger et al., 2016b, Harrisberger et al., 2016c) or in FEP-patients (Ho et al., 2017a, Ho et al., 2017b).

Vertex analyses revealed widespread alterations in FEP-patients while in the CHR-P group, no significant differences were observed. Consistent with our findings, previous studies have found evidence for volumetric reductions in hippocampal subfields bilaterally in early psychosis, specifically CA2/3 and the subiculum (Baglivo et al., 2018, Vargas et al., 2018). On the other hand, illness progression has been associated with volumetric decline in CA1, CA2/3, DG, and (pre-) subiculum bilaterally (Vargas et al., 2018).

In contrasting with our data, however, others (e. g. Sasabayashi et al., 2021) have identified shared deficits between CHR-Ps and schizophrenia patients in CA1 as well as in the hippocampal tail. Accordingly, further longitudinal data will be required to determine the precise trajectory of hippocampal shape abnormalities from the CHR-P state to manifest psychosis and schizophrenia.

In the present study, CHR-Ps and FEPs showed an overlapping and specific deficit in hippocampal volume, providing support for the hypothesis that hippocampal dysfunctions may constitute a core signature of early-stage psychosis (e.g. Lieberman et al., 2018). Importantly, participants with substance abuse and affective disorders were not characterized by hippocampal volume loss, suggesting that the observed reductions may be specifically related to psychosis and not to other comorbid psychopathology (e.g. Cole et al., 2011, Santos et al., 2018). In addition, the hippocampal deficits in CHR-P and FEP-groups were not influenced by antipsychotic and antidepressant medication status.

The overlapping volumetric reductions in the hippocampus in both FEP and CHR-P groups indicate a potential role for hippocampal alterations in development of psychosis. However, the more pronounced hippocampal dysfunctions in both volume and shape in the FEP group suggest stage-specific differences that raises questions regarding the underlying mechanisms and origins. One possibility is that hippocampal dysfunctions are the result of prolonged psychosis and associated changes in hippocampus physiology involving elevated glutamatergic neurotransmission as previously proposed (e.g. Lieberman et al., 2018, Plitman et al., 2014). In addition, antipsychotic medication levels have been related to GM loss in schizophrenia (van Haren et al., 2008) as well as hippocampal shape changes (Gutman et al., 2022).

Finally, hippocampal deficits have been also found in individuals at high genetic risk (Ganzola, Maziade & Duchesne, 2014) as well as unaffected relatives of individuals with psychosis (e.g. Boos et al., 2007, Choi et al., 2022). Accordingly, it is conceivable that hippocampal abnormalities are driven partially by genetic susceptibility. To distinguish between these possibilities, further longitudinal studies are required in CHR-Ps and FEPs to identify the trajectory and contribution of anatomical and functional hippocampal alterations towards the development of psychosis as well as potential subgroups with distinct genetic contributions.

Several limitations must be considered in the interpretation of our findings. Firstly, hippocampal volume deficits in the CHR-P group did not reach statistical significance following corrections for multiple comparisons. Secondly, the number of transitions to FEP was too small to properly assess the relationship with hippocampal alterations.

## 5 Summary

Our study shows that CHR-P and FEP groups were characterized by a specific and overlapping deficit in hippocampal anatomy which was not observed in other subcortical structures, highlighting the importance of abnormalities in the hippocampus for understanding early stage-psychosis. However, volumetric abnormalities were not related to clinical and functional outcomes in CHR-P participants, suggesting that other biomarkers may be more promising for predicting clinical trajectories. Future studies should employ multi-modal neuroimaging approaches to characterize the functional consequences of abnormal hippocampus anatomy during early-stage psychosis.

## 6 Contributions

PJU is the principal investigator for the YouR study. PJU, SL and AG contributed to the conception and design of the study. PJU, RK, RG, MS and FSL contributed to the data collection. GB, PJU and AF analysed the data. GB, AF and PJU drafted the manuscript, with critical revision from SL, JG and FSL. All authors contributed to the interpretation of data, and revised the manuscript. All authors are responsible for the reported research, and have approved the manuscript as submitted.

All authors are responsible for the reported research, and have approved the manuscript as submitted.

## Declaration of Competing Interest

The authors declare the following financial interests/personal relationships which may be considered as potential competing interests: PJU has received research support from Lilly and Lundbeck outside the submitted work. SML has received a personal fee from Sunovion outside the submitted work. All other authors report no biomedical financial interests or potential conflicts of interest.
