# Supplementary material for: Hippocampal structural alterations in early-stage psychosis: Specificity and relationship to clinical outcomes
Source: Neuroimage Clin. 2022 Jun 16;35:103087. doi: 10.1016/j.nicl.2022.103087 (PMC9421451; doi:10.1016/j.nicl.2022.103087)
Supplement: Supplementary data 1 [file mmc1.docx]

Supplementary Table 1: Subcortical volumes and linear model analyses

| Subcortical Volumes (mean mm, SD) | | | | |
| --- | --- | --- | --- | --- |
|  | HC | CHR-N | CHR-P | FEP |
| Amygdala | 1286 (219.2) | 1275 (203.3) | 1272 (197.1) | 1246 (186.4) |
| Caudate | 3822 (415.2) | 3767 (434.7) | 3750 (412) | 3823 (475.1) |
| Hippocampus | 4069 (328.5) | 4108 (366.9) | 3908 (428.7) | 3892 (416.8) |
| Nucleus Accumbens | 533.9 (87.72) | 514.4 (84.79) | 533 (77.47) | 540.1 (96.8) |
| Pallidum | 1801 (194.8) | 1762 (143.7) | 1763 (178.9) | 1852 (181.8) |
| Putamen | 5380 (521.7) | 5197 (467.6) | 5205 (552.3) | 5440 (590.7) |
| Thalamus | 8144 (699.1) | 8062 (617) | 7979 (744.7) | 8169 (737.3) |
|  |  |  |  |  |
| **Subcortex Linear Models, hemispheres averaged** (t, p) | | | | |
|  | | | | |
| Amygdala | 16.71 (< 0.001) | 0.17 (0.863) | -0.08 (0.939) | -1.9 (0.059) |
| Caudate | 30.8 (< 0.001) | -0.24 (0.813) | -1.48 (0.140) | -1.04 (0.300) |
| Hippocampus | 29.36 (< 0.001) | 1.22 (0.225) | -2.38 (0.018) | -3.75 (<0.001) |
| Nucleus Accumbens | 17.54 (< 0.001) | -0.73 (0.46) | 0.28 (0.78) | -0.64 (0.53) |
| Pallidum | 35.05 (< 0.001) | -0.49 (0.624) | -1.28 (0.202) | -0.09 (0.930) |
| Putamen | 33.67 (< 0.001) | -1.36 (0.176) | -2.4 (0.017) | -0.78 (0.433) |
| Thalamus | 44.36 (< 0.001) | 0.41 (0.682) | -1.43 (0.153) | -2.25 (0.025) |
|  |  |  |  |  |
| **Subcortex Linear Models, left hemispere** (t, p) | | | | |
|  | HC [intercept] | CHR-N | CHR-P | FEP |
| Amygdala | 13.65 (<0.001) | 1.18 (0.239) | 0.42 (0.678) | -1.88 (0.061) |
| Caudate | 30.02 (<0.001) | 0.16 (0.873) | -1.01 (0.314) | -1.35 (0.179) |
| Hippocampus | 27.15 (<0.001) | 0.57 (0.566) | -2.69 (0.008) | -3.69 (<0.001) |
| Nucleus Accumbens | 13.78 (<0.001) | -1.11 (0.27) | -0.28 (0.78) | -0.55 (0.58) |
| Pallidum | 31.35 (<0.001) | -0.36 (0.72) | -0.50 (0.61) | 0.38 (0.71) |
| Putamen | 32.78 (<0.001) | -1.81 (0.071) | -2.51 (0.013) | -0.60 (0.549) |
| Thalamus | 44.36 (<0.001) | 0.41 (0.682) | -1.43 (0.153) | -2.25 (0.025) |
|  |  |  |  |  |
| **Subcortex Linear Models, right hemisphere** (t, p) | | | | |
|  | HC [intercept] | CHR-N | CHR-P | FEP |
| Amygdala | 14.99 (<0.001) | -0.98 (0.330) | -0.59 (0.556) | -1.34 (0.182) |
| Caudate | 29.08 (<0.001) | -0.61 (0.541) | -1.83 (0.069) | -0.65 (0.518) |
| Hippocampus | 25.36 (<0.001) | 1.60 (0.112) | -1.58 (0.116) | -3.02 (0.003) |
| Nucleus Accumbens | 17.03 (<0.001) | -0.18 (0.86) | 0.78 (0.44) | -0.56 (0.25) |
| Pallidum | 33.67 (<0.001) | -0.56 (0.577) | -1.91 (0.057) | -0.57 (0.567) |
| Putamen | 32.61 (<0.001) | -0.82 (0.411) | -2.15 (0.033) | -0.92 (0.356) |
| Thalamus | 43.43 (<0.001) | 0.78 (0.438) | -1.00 (0.319) | -1.83 (0.069) |
|  |  |  |  |  |

| **Subcortex Linear Models (additional covariates), left hemispere** (t, p) | | | | | | | | |
| --- | --- | --- | --- | --- | --- | --- | --- | --- |
|  | HC [intercept] (N=48) | CHR-N (N=35) | CHR-P (N=107) | FEP (N=13) | TBV | Age | Education | Handedness (right/left ratio) |
| Amygdala | 11.57 (<0.001) | -0.19 (0.853) | 0.40 (0.690) | -0.77 (0.444) | 5.79 (<0.001) | -0.15 (0.883) | 1.99 (0.048) | 1.52 (0.131) |
| Caudate | 25.16 (<0.001) | -0.16 (0.875) | -0.63 (0.532) | 1.49 (0.138) | 8.68 (<0.001) | -2.16 (0.032) | 0.36 (0.720) | 1.10 (0.271) |
| Hippocampus | 20.83 (<0.001) | 1.05 (0.294) | -1.82 (0.070) | -1.86 (0.065) | 6.40 (<0.001) | 1.27 (0.206) | 0.67 (0.504) | 1.69 (0.092) |
| Nucleus Accumbens | 14.33 (<0.001) | -0.44 (0.658) | 0.70 (0.486) | 0.95 (0.343) | 7.20 (<0.001) | -0.61 (0.542) | 1.66 (0.098) | 0.55 (0.582) |
| Pallidum | 27.51 (<0.001) | -0.64 (0.525) | -0.15 (0.883) | 0.79 (0.432) | 12.84 (<0.001) | -0.72 (0.470) | 2.24 (0.026) | 0.88 (0.382) |
| Putamen | 29.74 (<0.001) | -1.74 (0.083) | -1.62 (0.106) | 0.80 (0.424) | 12.50 (<0.001) | -1.22 (0.225) | 1.37 (0.173) | 1.26 (0.208) |
| Thalamus | 38.66 (<0.001) | 0.86 (0.390) | -0.49 (0.622) | -0.09 (0.928) | 17.31 (<0.001) | 0.49 (0.627) | 1.75 (0.081) | 1.61 (0.110) |
|  |  |  |  |  |  |  |  |  |
| **Subcortex Linear Models additional covariates), right hemisphere** (t, p) | | | | | | | | |
|  | HC [intercept] | CHR-N | CHR-P | FEP | TBV | Age | Education | Handedness |
| Amygdala | 11.48 (<0.001) | 0.51 (0.61) | -0.15 (0.88) | -0.43 (0.67) | 4.10 (<0.001) | 0.70 (0.49) | 0.28 (0.78) | 0.06 (0.95) |
| Caudate | 25.68 (<0.001) | -0.51 (0.61) | -1.16 (0.25) | -0.86 (0.39) | 9.25 (<0.001) | -1.20 (0.23) | -0.18 (0.86) | 0.40 (0.69) |
| Hippocampus | 22.86 (<0.001) | 0.57 (0.568) | -2.28 (0.024) | -1.85 (0.066) | 6.88 (<0.001) | 0.73 (0.463) | 0.19 (0.849) | 2.23 (0.027) |
| Nucleus Accumbens | 13.55 (<0.001) | -0.78 (0.44) | 0.32 (0.75) | 0.95 (0.34) | 6.70 (<0.001) | 0.36 (0.72) | -0.14 (0.88) | 1.63 (0.10) |
| Pallidum | 29.52 (<0.001) | -0.19 (0.85) | -0.79 (0.43) | 1.54 (0.13) | 14.06 (<0.001) | -0.06 (0.95) | 1.64 (0.10) | 0.69 (0.49) |
| Putamen | 29.49 (<0.001) | -1.15 (0.252) | -1.95 (0.053) | 0.66 (0.511) | 12.54 (<0.001) | -1.28 (0.204) | 1.24 (0.217) | 1.09 (0.276) |
| Thalamus | 37.42 (<0.001) | 0.86 (0.39) | -0.47 (0.64) | -0.03 (0.98) | 15.98 (<0.001) | 0.49 (0.63) | 1.45 (0.15) | 1.65 (0.10) |

*Note.* p-values shown are uncorrected. Sample sizes for the additional covariate analysis are smaller due to missing data.
