# Supplementary material for: Hippocampal structural alterations in early-stage psychosis: Specificity and relationship to clinical outcomes
Source: Neuroimage Clin. 2022 Jun 16;35:103087. doi: 10.1016/j.nicl.2022.103087 (PMC9421451; doi:10.1016/j.nicl.2022.103087)
Supplement: Supplementary data 2 [file mmc2.docx]

Supplementary Table 2: Correlations between hippocampal volumes and clinical severity, functioning and cognitive performance

| **Correlations with hippocampal volume** (CHR-P group) | | | | |
| --- | --- | --- | --- | --- |
|  | Right hippocampus r | p | Left hippocampus r | p |
| Total CAARMS severity | 0.083 | 0.389 | 0.082 | 0.394 |
| CAARMS UTC | 0.035 | 0.716 | 0.067 | 0.486 |
| CAARMS NBI | 0.031 | 0.743 | -0.006 | 0.953 |
| CAARMS PA | 0.095 | 0.320 | 0.019 | 0.841 |
| CAARMS DS | 0.117 | 0.223 | 0.191 | 0.117 |
| Total SPI-A severity | 0.120 | 0.203 | 0.082 | 0.394 |
| GAF 0 | -0.141 | 0.140 | -0.089 | 0.345 |
| GAF 6m | -0.118 | 0.266 | 0.069 | -0.118 |
| GAF 12m | 0.088 | 0.452 | 0.282 | 0.013 |
| BACS composite score | -0.022 | 0.859 | 0.046 | 0.703 |
| BACS verbal fluency | -0.022 | 0.857 | 0.046 | 0.704 |
| BACS working memory | -0.035 | 0.771 | -0.034 | 0.779 |
| BACS ToL | -0.022 | 0.858 | 0.046 | 0.704 |
| BACS motor speed | -0.022 | 0.858 | 0.046 | 0.703 |
| BACS symbol coding | -0.021 | 0.859 | 0.046 | 0.703 |

BACS, Brief Assessment of Cognition in Schizophrenia; CAARMS, Comprehensive Assessment of At Risk Mental States; HC, healthy controls; CHR-N, clinical risk-negative; CHR-P, clinical high-risk positive; FEP, first-episode psychosis; GAF, global assessment of functioning; SPI-A, Schizophrenia Proneness Instrument, Adult version; SD, standard deviation of the mean; AD, antidepressant; AP, antipsychotic

Note. P-values are shown without correction.
