# Supplementary material for: Hippocampal structural alterations in early-stage psychosis: Specificity and relationship to clinical outcomes
Source: Neuroimage Clin. 2022 Jun 16;35:103087. doi: 10.1016/j.nicl.2022.103087 (PMC9421451; doi:10.1016/j.nicl.2022.103087)
Supplement: Supplementary data 3 [file mmc3.docx]

Supplementary Table 3: Hippocampal shape analysis

| **Hippocampal shape analysis** | | | | |
| --- | --- | --- | --- | --- |
|  | Peak F | p | Peak coordinates (x, y ,z)* |  |
| HC vs CHR-P |  |  |  |  |
| right | 25.050 | 0.070 NS |  |  |
| left | 8.120 | 0.530 NS |  |  |
| HC vc CHR-N |  |  |  |  |
| right | 8.530 | 0.290 NS |  |  |
| left | 10.360 | 0.170 NS |  |  |
| HC vs FEP |  |  |  |  |
| right | 16.140 | **<0.01** | 116, 105, 50 |  |
| left | 18.910 | **<0.001** | 62, 106, 50 |  |
| CHR-P vs FEP |  |  |  |  |
| right | 10.520 | 0.230 NS |  |  |
| left | 19.910 | 0.010 NS | 120, 103, 61 |  |
