# Supplementary material for: Hippocampal structural alterations in early-stage psychosis: Specificity and relationship to clinical outcomes
Source: Neuroimage Clin. 2022 Jun 16;35:103087. doi: 10.1016/j.nicl.2022.103087 (PMC9421451; doi:10.1016/j.nicl.2022.103087)
Supplement: Supplementary data 4 [file mmc4.docx]

Supplementary Table 4: Effects of antidepressant use on subcortical volumes in the CHR-P group

| **Antidepressant effects on subcortical volumes** | | | | |
| --- | --- | --- | --- | --- |
|  | t | p |  |  |
| AD vs no AD (CHR-P) |  |  |  |  |
| Amydgala |  |  |  |  |
| right | -1.155 | 0.251 |  |  |
| left | -1.455 | 0.149 |  |  |
| Caudate |  |  |  |  |
| right | 1.549 | 0.124 |  |  |
| left | 1.119 | 0.266 |  |  |
| Hippocampus |  |  |  |  |
| right | 0.097 | 0.923 |  |  |
| left | 0.638 | 0.525 |  |  |
| Nucleus Accumbens |  |  |  |  |
| right | -0.710 | 0.479 |  |  |
| left | 0.525 | 0.601 |  |  |
| Pallidum |  |  |  |  |
| right | -0.342 | 0.733 |  |  |
| left | -0.749 | 0.455 |  |  |
| Putamen |  |  |  |  |
| right | -0.106 | 0.916 |  |  |
| left | -0.189 | 0.850 |  |  |
| Thalamus |  |  |  |  |
| right | -0.943 | 0.348 |  |  |
| left | -0.660 | 0.510 |  |  |
