# Supplementary material for: Hippocampal structural alterations in early-stage psychosis: Specificity and relationship to clinical outcomes
Source: Neuroimage Clin. 2022 Jun 16;35:103087. doi: 10.1016/j.nicl.2022.103087 (PMC9421451; doi:10.1016/j.nicl.2022.103087)
Supplement: Supplementary data 5 [file mmc5.docx]

Supplementary Table 5: Effects of antipsychotic use on subcortical volumes in the FEP group

| AP vs no AP (FEP) |  |  |  |  |
| --- | --- | --- | --- | --- |
| Amydgala |  |  |  |  |
| right | 1.334 | 0.205 |  |  |
| left | -0.240 | 0.814 |  |  |
| Caudate |  |  |  |  |
| right | 0.486 | 0.635 |  |  |
| left | 1.273 | 0.225 |  |  |
| Hippocampus |  |  |  |  |
| right | 1.240 | 0.243 |  |  |
| left | 0.654 | 0.524 |  |  |
| Nucleus Accumbens |  |  |  |  |
| right | 0.157 | 0.878 |  |  |
| left | -0.229 | 0.820 |  |  |
| Pallidum |  |  |  |  |
| right | 0.295 | 0.772 |  |  |
| left | 0.270 | 0.792 |  |  |
| Putamen |  |  |  |  |
| right | -0.509 | -0.620 |  |  |
| left | -0.125 | 0.903 |  |  |
| Thalamus |  |  |  |  |
| right | -0.129 | 0.899 |  |  |
| left | -0.314 | 0.759 |  |  |

Note: Data only available for 15 FEP subjects
