# Supplementary material for: Hippocampal structural alterations in early-stage psychosis: Specificity and relationship to clinical outcomes
Source: Neuroimage Clin. 2022 Jun 16;35:103087. doi: 10.1016/j.nicl.2022.103087 (PMC9421451; doi:10.1016/j.nicl.2022.103087)
Supplement: Supplementary data 6 [file mmc6.docx]

Supplementary Table 6: Relationship between GAF scores and hippocampal volumes in the CHR-P group

| **Relationship between hippocampal volumes and functional outcomes** | | | | |
| --- | --- | --- | --- | --- |
|  | Z (binomial GLM) | p |  |  |
| Baseline GAF | -0.550 | 0.580 |  |  |
| GAF (6m) | 0.980 | 0.330 |  |  |
| GAF (12m) | 2.510 | 0.012 |  |  |
